# Total Pelvic Exenteration for Gynecologic Malignancies

**DOI:** 10.1155/2012/693535

**Published:** 2012-06-10

**Authors:** Elisabeth J. Diver, J. Alejandro Rauh-Hain, Marcela G. del Carmen

**Affiliations:** Division of Gynecologic Oncology, Vincent Obstetrics and Gynecology, Massachusetts General Hospital, Harvard Medical School, 55 Fruit Street, Yawkey 9E Boston, MA 02114, USA

## Abstract

Total pelvic exenteration (PE) is a radical operation, involving en bloc resection of pelvic organs, including reproductive structures, bladder, and rectosigmoid. In gynecologic oncology, it is most commonly indicated for the treatment of advanced primary or locally recurrent cancer. Careful patient selection and counseling are of paramount importance when considering someone for PE. Part of the evaluation process includes comprehensive assessment to exclude unresectable or metastatic disease. PE can be curative for carefully selected patients with gynecologic cancers. Major complications can be seen in as many as 50% of patients undergoing PE, underscoring the need to carefully discuss risks and benefits of this procedure with patients considering exenterative surgery.

## 1. Introduction

Pelvic exenteration (PE) describes a radical surgery involving the en bloc resection of the pelvic organs, including the internal reproductive organs, bladder, and rectosigmoid. Indications include advanced primary or recurrent pelvic malignancies, most commonly centrally recurrent cervical carcinoma, but also other gynecologic tumors and urologic and rectal cancers. Distant metastasis has traditionally been a contraindication to PE with curative intent. As the best chance for disease-free survival is surgical resection of regional disease, this procedure is an opportunity to cure advanced and recurrent cancers confined to the pelvis. PE has also been used for palliation of symptoms related to radiation necrosis or extensive tumor burden. Both total and partial PE require extensive reconstruction and surgical recovery with significant associated morbidity and mortality. Careful patient selection is required to balance the potential goal of cure or symptom palliation with surgical risk.

The first cases of total PE were described by Brunschwig in 1948 as a palliative procedure for symptoms caused by locally advanced gynecologic cancers. This demonstrated proof of concept for PE, with a postoperative survival of up to 8 months, and a 23% surgical mortality rate [1]. Subsequent data demonstrated that the technique could offer a chance of cure for centrally located tumors, not just palliation, and the focus of the surgery shifted to one of curative intent. Various surgical approaches both for sparing uninvolved pelvic organs and removing extraperitoneal structures such as the sacrum were attempted. Major breakthroughs included separate stomata for urine and fecal diversion and the use of omentum to protect the empty and denuded pelvic space and reduce abscess formation and intestinal obstruction [2, 3]. More recently, techniques to resect tumor involving the pelvic sidewall, previously a contraindication to PE, have been described offering more patients a chance at curative surgery [4]. PE may also be combined with intra-operative radiation therapy for improved disease control at the pelvic sidewall or possible positive margins [5, 6].

Since 1948 several developments in perioperative care and surgical technique have improved survival, morbidity, and mortality, with recent mortality rates quoted <5%. Development of continent urinary conduits and orthotopic neobladders, as well as low rectal anastomoses has led to the completion of PE without formation of stomata [7]. Various techniques for functional neovaginas have been described, allowing patients to maintain sexual function if they desire. Advances in laparoscopic and robotic assisted technology applied to PE have improved operative recovery. Despite these significant advances and five-year survival rates of approximately 50%, PE remains a radical procedure with significant complications (31–92%; see Table 1), both physical and psychological [8]. Given the nature of this procedure, appropriate patient selection and counseling remain paramount.

## 2. Indications and Outcomes

### 2.1. Cervical Cancer

Traditionally PE has been used for centrally recurrent cervical carcinoma, both squamous and adenocarcinoma, with well-documented salvage potential. Up to 25% of women with FIGO stage IB-IIA cervical cancer may recur after initial therapy [9]. Frequently, these recurrences may be treated with radiotherapy; however, radical surgery may offer an alternative for curative treatment. Survival rates ranging from 16 to 60% are reported for these patients [10, 11]. Long-term survival is directly correlated with complete tumor resection [12, 13], so establishing resectability is a key aspect of preoperative planning. Time from primary treatment, with radiation or chemoradiation, to time of PE has also been shown to be related to survival and disease-free interval [12], with women requiring PE for recurrence less than 2 years following primary therapy demonstrating an 8-month survival versus 33 months in women who recurred more than 2 years following initial treatment in one study [14], though this has not been shown in all series [10]. PE has also been utilized as a potentially curative primary treatment for locally advanced cervical cancer (FIGO stage IVa), a practice exercised more frequently in Germany than the United States [15]. For example, in their series, Marnitz et al. reported a 52.5% five-year survival [12].

### 2.2. Uterine Cancer

Cases of PE for a variety of histologic types of uterine cancer have been reported, with outcomes similar to PE for other indications. Most recurrent uterine cancers spread beyond the pelvis, given their propensity for diffuse abdominal or heterogenous spread, making PE appropriate intervention for only a select group of patients with recurrent uterine malignancies. Women with only loco-regional recurrence, however, may be candidates for PE with curative intent. Khoury-Collado et al. [16] described a series of 21 women with recurrent uterine cancers who underwent PE and demonstrated a five-year survival of 40%. The study also noted varying outcomes dependent on histology, with endometrioid adenocarcinoma (50% five-year survival rate) and sarcoma (66% five-year survival rate) demonstrating improved survival over a group of women with tumors with serous, mixed, and carcinosarcoma-histology (14% five-year survival rate). Morris et al. [17] reported a five-year survival rate of 45% following PE for recurrent endometrial cancer. Given the similarity of complication rates (48–60%) and survival to PE for cervical cancer, patients with locally recurrent uterine cancer may be considered candidates for the procedure.

### 2.3. Vulvar Cancer

Vulvar cancer has a propensity for regional metastases. For patients with advanced primary or recurrent vulvar cancer who do not have the option of treatment with radiation therapy, PE may be appropriate. Forner and Lampe [18] published a series of 27 patients undergoing PE. The authors demonstrated results similar to other gynecologic malignancies, with a five-year survival of 62%. Complete resection with no evidence of residual disease was associated with improved outcomes, a five-year survival rate of 74%, compared to 21% in patients without complete resection. Absence of tumor lymph node invasion was also associated with an improved five-year survival rate (83% versus 36%). In contrast, combination therapy with vulvectomy and radiotherapy has been described for locally advanced vulvar cancer with the goal to spare the pelvic organs, with five-year survival in two series of 45% and 72%, and sparing of the pelvic organs in 62.5% and 89% of patients [19, 20].

### 2.4. Ovarian Cancer

Given the propensity of ovarian cancer to spread throughout the abdomen, women with this disease are rarely candidates for PE with curative intent. Supralevator PE has been reported when needed for optimal cytoreduction, combined with standard staging procedures and for recurrent disease. Two series of modified posterior PE for ovarian cancer demonstrated median survival 33 and 37.4 months after initial surgery. Optimal cytoreduction was achieved in 46% and 58% of patients in the series [21, 22], demonstrating this technique may be used to achieve optimal cytoreduction in patients with disease requiring rectosigmoid resection.

### 2.5. Vaginal Cancer

As vaginal cancer is rare, this review could not identify any literature specifically addressing PE for this indication. Several cases of vaginal cancer, both primary and recurrent, undergoing PE have been included in larger studies, most frequently including the results for these patients combined with results for cervical cancer [10, 23]. It may be hypothesized, that results following PE for vaginal carcinoma would be similar to those for cervical cancer provided the same other parameters for patient selection apply.

### 2.6. Palliative PE

PE has been described for palliation rather than for curative intent, most frequently in the setting of severe radiation necrosis. Indications have also included intractable hemorrhage due to tumor invasion and fistulae. Both morbidity and mortality were shown to be higher in this group of patients as opposed to those undergoing PE with curative intent, though improvements in quality of life are reported [24, 25]. PE is thus only considered for palliation if there is no reasonable alternative, though with the development of minimally invasive surgical technology, PE may become a more feasible option [26].

## 3. Patient Selection and Preoperative Screening

PE is a major surgery with significant morbidity, and as such selecting appropriate patients is essential. If the surgery is undertaken with curative intent, the tumor should be fully resectable with negative margins. Recurrence should be biopsy-proven. Classically, disease burden was required to be limited to the central pelvis, but with new surgical developments candidates for curative PE may now also include patients with positive lymph nodes, pelvic sidewall involvement, and local bone invasion.

Regardless of the indication, patients undergoing PE must be in otherwise good medical health to be able to tolerate a long surgical procedure with extensive fluid shifts and prolonged hospital stay. Major medical comorbidities may be a potential contraindication to PE. Preoperative evaluation includes a complete history, physical exam, and, if necessary, an exam under anesthesia, biopsy of any suspicious lesion such as an enlarged lymph node, evaluation of specific patient concerns suggesting metastatic spread, such as unilateral leg pain, chest radiograph or computed tomography (CT). In general, cystoscopy and sigmoidoscopy are not necessary unless the bladder or rectum is to be spared. In this case, careful evaluation of these structures is imperative to rule out occult metastatic disease.

Laboratory tests should include a complete blood count, platelet count, comprehensive metabolic panel, including hepatic and renal function, as well as clotting factors and urinalysis. Elevated liver function tests require further evaluation to rule out liver metastasis. Patients with a bleeding diathesis and any anemia should have their anemia corrected preoperatively. Any infectious process should be fully evaluated and whenever possible resolved preoperatively. Patients with underlying diabetes should have their glucose control optimized before PE. Patients should be offered testing for human immunodeficiency virus, which may be a contraindication to PE.

Patients being considered for pelvic PE need to be carefully counseled. Given the nature of the surgery, patients should be counseled about changes in body image and function. Specifically, patients should have an understanding of anatomical changes involving creation of colostomy and urinary conduit and need to be accepting of major changes in body image even in the setting of reconstructive surgery. Patients require significant family support, intact mental capacity and access to continued and long-term medical care. We recommend sharing printed literature and illustrations depicting ostomies and conduits, as well as offering patients the opportunity to speak with other women who have undergone the procedure. Patients should meet with ostomy nursing staff to begin the education process preoperatively and gain confidence with management of the ostomy and conduits. During these visit, patients can be marked for optimal placement of the ostomy and conduit. Part of the counseling sessions should focus on sexual function and how this will change for both patients choosing to have creation of a neovagina, as well as for those declining this part of the reconstruction. A formal psychiatric consultation may be appropriate for some patients.

Patients should be informed of all possible perioperative complications, including infectious, thromboembolic, gastrointestinal, urinary, psychiatric, readmission, and reoperation. Women being considered for PE should be informed of a 3–5% risk of operative mortality. Importantly, as noted by Khoury-Collardo et al. [16] some of these complications occur more frequently in the remote postoperative period (days 31–90) than in the immediate one (0–30 days). Patients should expect frequent visits to the hospital during this time given the risk not only of immediate but also delayed complications. Of note, part of the preoperative counseling should include the impact aborting the operation for unexpected surgical findings may have on the patient. That is, patients should be informed that even with the use of state-of-the art, preoperative imaging, the possibility of finding metastatic disease continues to exist, and a minority of exenterative procedures are aborted at the time of surgical exploration. It is critical that patients have sufficient medical and emotional support to manage the physical and psychological challenges central to the operation [27].

### 3.1. Imaging

The presence of metastasis outside the pelvis is an absolute contraindication to PE. Therefore, the goal of diagnostic techniques is to find evidence of unresectable or metastatic disease; thus, making the woman an unsuitable candidate for PE. A number of diagnostic techniques can aid in assessing unresectable disease in a patient who is believed to have central pelvic disease. Computed tomography (CT) and magnetic resonance imaging (MRI) can be helpful in assessing the presence of lateral pelvic wall invasion or liver metastasis. However, major limitations of CT and MRI lie in their inability to assess minimally enlarged nodes to detect microscopic peritoneal disease and to distinguish fibrosis from tumor in recurrent disease, and the fact that most patients have usually received extensive radiation makes distinguishing radiation fibrosis from malignant tumor extremely difficult [28].

F-fluorodeoxyglucose positron emission tomography (FDG PET) has been shown to perform better in this population. The only prospective study to date in which all patients underwent surgical exploration with curative intent and in which almost all PET positive sites were biopsied showed that sensitivity and specificity of PET imaging in metastatic disease in patients being considered for PE was 100 and 73%, respectively. Despite a negative predictive value of 100%, positive predictive value was 55%. The high false positive rate found in this study makes surgery obligatory for all PE candidates [29]. Bone scans are usually not indicated unless there is a history of recent bone pain and concern for bony metastases.

### 3.2. Explorative Phase

The procedure begins with the patient in low lithotomy position to allow for abdominal and perineal portions of the surgery. Combined epidural and general anesthesia may be considered for additional postoperative pain control. PE is traditionally performed as an open abdominal procedure, but recent developments in laparoscopy and robotics have allowed for the minimally-invasive adaptation of the technique. Open technique will be described here. The abdomen is opened with a vertical midline incision to allow for maximum ability to explore the upper abdomen as well as the pelvis. The abdomen and pelvis are then thoroughly examined for evidence of metastatic disease. Washings may be sent for cytology. Any suspicious lesion is biopsied and sent for frozen section to exclude the possibility of distant metastatic disease that would preclude complete resection or alter the surgical plan. For recurrent cervical cancer, low para-aortic and pelvic lymph node dissection may be performed again to preclude metastatic spread beyond the pelvis. Lateral involvement of disease to the pelvic sidewall should be assessed at this time. Once the disease has been confirmed to be resectable, the operation may proceed [8, 30, 31].

The round ligaments are divided, and the paravesical and pararectal spaces are developed. At this time the pelvic sidewalls may again be examined. At this point, the extent of PE must be determined. Total PE includes removal of the bladder and distal ureters, portions of rectum and sigmoid colon, internal reproductive organs (if still present), and vagina. In well-selected patients, this procedure generally ensures complete negative margins from the tumor specimen. Occasionally, if the anatomic location of the recurrence is only the anterior or posterior compartment of the pelvis, the colon or bladder may be spared and only an anterior exenteration or posterior exenteration may be necessary.

### 3.3. En Bloc Resection

Removal of the specimen begins with the ligation and division of the fibrovascular pedicle containing the uterine vessels, cardinal ligament, and the ureter bilaterally. The uterine artery is ligated at is origin from the hypogastric, lateral to the ureter. The infundibulopelvic ligaments are ligated above the level of the common iliac vessels. The sigmoid is then mobilized and transected with a gastrointestinal anastomotic stapler (GIA), and the sigmoid vessels are identified and ligated. Care must be taken to preserve blood flow to the remaining colon—usually the sigmoid artery is left intact and the superior hemorrhoidal artery is ligated. The avascular plane between the sigmoid and the sacrum is developed to the level of the levator ani muscles. The prevesical space is extended bluntly. At this point, the specimen should be freely mobile in the pelvis.

The perineal portion of the procedure is then performed (or may be performed synchronously with an additional surgeon). An incision is marked to include the urethra, vaginal opening, anus, and possibly the vulva. The muscles of the pelvic floor are transected circumferentially. The pubococcygeal and anococcygeal ligaments are identified and divided. Upon completion of the dissection, the entire specimen is free to be removed.

### 3.4. Alternative Types of PE

#### 3.4.1. Anterior PE

Anterior PE involves the removal of the bladder and internal reproductive organs but spares the gastrointestinal tract. The rectosigmoid, anus, and lower portion of the posterior vagina are left intact. After division of the cardinal ligaments, uterine vessels, and ureters, the rectum is separated from the upper vagina. The rectum is retracted posteriorly by rectovaginal bimanual exam to ensure the space is clear of tumor and resectable. The uterosacral ligaments are dissected and divided. An incision is made into the peritoneum of the cul-de-sac, and the rectum is dissected sharply off the upper vagina. The incision into the posterior vagina at its midportion is made. Biopsies of the vagina or margins may be sent for frozen section.

#### 3.4.2. Posterior PE

Posterior PE removes the internal reproductive organs and the rectosigmoid but spares the anterior vagina, urinary bladder, and ureters. In previously irradiated pelves, it is important to consider the possibility of urinary fistulae developing following a posterior PE given the possibility for devascularization. The uterovesical peritoneum is incised after the paravesical and pararectal spaces have been developed. The ureters are identified and dissected as in a radical hysterectomy, and the uterine arteries and cardinal ligaments ligated. The anterior vagina is incised and dissected sharply. The perineal phase of the operation spares the urethra and the anterior vagina.

Modified posterior PE, as for cytoreduction in ovarian cancer, is a supralevator dissection. There is no perineal phase to the operation. If enough rectum remains (more than 6 centimeters), a low rectal anastomosis may be made, sparing the patient a stoma.

#### 3.4.3. Supralevator PE

If the tumor does not involve the vulva or lower third of the vagina, the patient may be a candidate for a supralevator PE [10]. After the specimen is mobilized as in a total PE, an incision is made into the posterior vaginal wall below the tumor, ensuring an adequate margin. The rectum is isolated and divided with a stapling device, leaving an anorectal stump and possibility for low rectal anastomosis.

### 3.5. Reconstruction

#### 3.5.1. Urinary Diversion

Brunschwig initially designed reconstruction after PE with an ureterosigmoidostomy, known as a wet colostomy, with urine and feces emptying through one stoma [1]. This was complicated by infection and patient dissatisfaction. Subsequently, Bricker developed the isolated ileal loop conduit [2]. In current practice, both incontinent ileal and colonic conduits are used, as well as a variety of continent urinary reservoirs, most commonly the Miami pouch [30, 31]. The standard ileal conduit is formed by an isolated segment of distal ileum with its vasculature. The ureters are anastomosed directly to the ileum at one end, and the other end is brought to the skin as a stoma. A drainage bag must be worn over the stoma.

The Miami pouch was first reported by Bejany and Politano in 1988, a modification of prior continent colonic pouches designed to reduce incontinence [32]. A segment of distal ileum and ascending colon are used for the pouch. The ileum is transected 10 to 15 centimeters proximal to the ileocecal valve, and the transverse colon is transected distal to the middle colic artery. An appendectomy is performed. To form the bulk of the pouch, the colon is opened along the tenia, and the open edges are approximated by folding the colon segment into a u-shape conduit and the edges closed with a stapling device. This formation of the colon creates a reservoir and interrupts the ability of the bowel to peristalse and increase the pouch pressure. For the ureteral anastomoses, the distal ends of the ureters are flayed and then sutured with fine, absorbable suture to the colonic submucosa. Ureteral stents are placed and secured. Attention is then turned to the ileum, which is tapered distally to support the ileocolic valve and prevent reflux. The free end of the ileum is brought to the skin surface as a stoma. The patient will be required to self-catheterize this stoma, but she is spared a drainage device if the procedure is successful. Penalver et al. [33] in a follow-up study of the Miami pouch reported 92% continence and reservoir volume of average 650 mL, allowing for a reasonable catheterization interval. Another recent series reported 89% of women were continent of urine [11].

#### 3.5.2. Fecal Diversion

For patients whose disease requires infralevator dissection posteriorly, permanent end colostomy will be required because the anal sphincter is compromised or excised. If the sphincter and enough rectum may be spared without compromising the chance at complete disease resection, low rectal anastomosis may be considered to restore continence. Direct end to end anastomosis with circular staplers is a reasonable option if enough healthy tissue remains. To improve frequency of stooling by improving the reservoir of the rectum, a colonic J-pouch may be used, particularly in patients with very little rectum remaining (less than 5 centimeters). Some authors, however, cite the frequency of recurrence of disease near the site of rectal anastomosis (45%) as a reason to perform complete resection and end colostomy in all patients [11]. Other authors strongly support low rectal anastomosis for a chance at preserved function and avoidance of undesirable colostomy for the patient [10].

#### 3.5.3. Neovagina

After vaginectomy, construction of a neovagina for restoration of sexual function should be offered to patients undergoing PE. Several options exist for the creation of a neovagina, including split-thickness skin grafts, myocutaneous grafts, and colon. Rectus abdominus myocutaneous (RAM) flaps have been reported routinely in the literature, with 93% viability in the series from UCLA [10]. The flap also serves to fill and vascularize the pelvic dead space.

The RAM flap may be harvested from the same midline vertical incision used for the PE, improving cosmetic outcomes for the patient. Consideration must be made in the selection of the flap, such as previous Maylard incision or other compromise to the inferior epigastric artery. A transverse or vertical flap may be constructed, at least 10 to 12 centimeters in length, maintaining the blood supply from the inferior epigastric artery. The flap is freed, elevated, and sutured into a tube with the skin at the interior, which will serve as the neovagina. The tube is then secured in the pelvis at the vaginal introitus. A mold with estrogen cream is left in the vagina to maintain the lumen for 5 to 7 days. The donor site is closed with the primary abdominal incision. Results are positive, with high flap viability [31]. Patient satisfaction and coitus rates are quoted as 58–78% [34, 35].

#### 3.5.4. Pelvic Floor Coverage

If a neovagina has been created with a myocutaneous flap, such as a RAM flap, this graft is usually sufficient to fill the pelvic dead space and ensure adequate vascularity. If no such procedure has been performed, it minimizes complications such as bowel obstruction and maximizes hemostasis to close the pelvic dead space. The most common mechanism for coverage is with the omental J-flap. The omentum is detached at the greater curvature of the stomach while preserving its origin containing the left gastroepiploic artery, which will supply the flap. The omentum is then brought into the pelvic dead space and sutured into place. Other options, such as mesh and pelvic packing, were attempted with poor outcomes [11].

## 4. Laparoscopic and Robotic-Assisted Surgery

Laparoscopic surgery has advanced considerably in recent years. The indications for its use have widened, and the superseding of open surgery seems inevitable in many areas of surgery. This revolution in surgery is in part associated with the technological advancement and a concomitant acquisition of advanced minimally invasive surgical skills by many gynecologic oncologists. Laparoscopy is now a well-accepted tool in the armamentarium of the treatment of gynecological cancer, and data have been published by various centers [36–39]. Minimal invasive surgery is generally associated with less intraoperative blood loss, postoperative pain, and shorter hospital stay.

Pomel et al. [40, 41] were the first group to report two cases of laparoscopic PE for gynecological cancer. The authors demonstrated in these reports the feasibility of this procedure. Both patients enjoyed the other well-known advantages of laparoscopy including minimal blood loss and quick ambulation, all contributing to a better postoperative quality of life. Subsequently, Lin et al. [42] reported a case of laparoscopy-assisted transvaginal total PE. In addition, Ferron et al. [43] published a series of five patients that underwent a laparoscopic assisted vaginal PE. Their series reports the first application of a rational combination of laparoscopic, perineal, and hand-assisted surgery, with the goal of limiting the potentially long laparoscopic time to a strict minimum. Of note, the authors elected to perform a hand-assisted Miami pouch through a minilaparotomy (5 cm) in order to reduce the operative time, safely perform the ureteral anastomosis, restore bowel continuity and, in addition, build the omental cylinder for vaginal reconstruction. The use of a perineal or vaginal approach allowed to quickly and safely free the specimen well above the pelvic floor. In a subsequent report by the same authors, with a mean follow-up of 14 months, four patients died of the disease (three were metastatic), one patient presented a local recurrence, and two patients are disease free [44].

Puntambekar et al. [45] reported in a series of 16 consecutive patients, the technique, feasibility, and safety of laparoscopic anterior PE as primary treatment for locally advanced pelvic cancers. Thirteen patients underwent anterior PE with ureterosigmoidostomy, while two patients required total PE with wet colostomy. The authors described a low rate of morbidity in their series. Two patients suffered from subacute intestinal obstruction and were treated conservatively. One patient had a ureteric leak that resolved with conservative management. Of note, after a mean follow up of 15 months, all patients were disease free. Puntambekar et al. [46] also described the feasibility of doing a laparoscopic total PE for palliation in advanced cervical cancer. Of the 7 patients included in their series, no patients required conversion to open surgery. The mean postoperative hospital stay was 8 (7–21) days. The mean followup was 11 (4–24) months and mean symptom free period was 8 (3–24) months. There was no major and unanticipated postoperative morbidity. There was no immediate postoperative mortality. In all patients, the pathology specimen had tumor free margins. The mean followup of the patients was 11 months (range 4 to 24 months); and the mean symptom free survival period was 8 months (range 3 to 24 months). Four patients subsequently died secondary to distant metastases. Three patients are now disease-free for more than a year.

The development of robotic technology has facilitated the application of minimally invasive techniques for the treatment and evaluation of patients with gynecological cancers. Robotic surgery offers several advantages over laparoscopy: a three-dimensional vision system, wristed instrumentation, and ergonomic positioning for the surgeon while performing surgical procedures. The enhanced visualization gives the gynecologic surgeon an improved ability to identify tissue planes, blood vessels, and nerves while performing the surgical procedure [47–49].

Since the first report of robotic-assisted radical hysterectomy by Sert and Abeler in 2006 for cervical cancer, there have been some reports of robotic-assisted laparoscopic PE [50–52]. The first cases of robotic-assisted laparoscopic PE were described by Pruthi et al. [53] in 12 women for clinically localized bladder cancer. Urinary diversion was performed extracorporeally (9 ileal conduit diversion, 3 orthotopic neobladder). Lim [54] reported the first case report of robotic assisted total PE with an ileal loop urinary diversion and an end colostomy for treatment of recurrent cervical cancer. Subsequently, Lambaudie et al. reported a case series of three patients that underwent robotic assisted total PE. Of note, the urinary diversion was made extracorporeally by a transrectal laparotomy. The authors reported that concerning hospital stay, there was no benefit comparing to laparotomy, essentially due to urinary diversion management (catheterization) and to self catheterization patient's autonomy.

Despite the apparent encouraging early results suggesting an advantage of minimally invasive surgery for PE, questions remain about the surgical effectiveness of this approach. Further study of minimally invasive techniques to perform a PE is needed prior to widespread clinical application of these techniques.

## 5. Complications

As it is a radical surgery performed in the setting of advanced tumor growth and frequently on irradiated tissue, PE is associated with a significant rate of complications, quoted about 40–50% for major complications and about 80% for minor complications. Mortality is quoted from 1–16%, with disparate causes including sepsis, thromboembolic disease, and cardiopulmonary failure. Despite significant advances in the last fifty years, the extensive nature of the surgery, including blood loss, fluid shifts, and operative time, have led to unavoidable risks. Infection is the most frequent morbidity (19–86%), with urinary infections and wound infections most commonly reported. Anastomotic leaks and fistulae from either diverting system are also relatively frequent, cited at 8–36%. Small bowel and ureteral obstructions also occur in about 5–10% of patients. Most of these complications can be managed conservatively, but significant numbers of patients require operative revision [10, 11, 55]. Death in the perioperative period occurs in fewer than 5 percent of patients, with women over the age of 65 at highest risk [10].

## 6. Postoperative Period

Given the radical and prolonged nature of this procedure, patients and providers must be prepared for a long and potentially complicated hospital course. Many patients require a stay in the intensive care unit immediately postoperatively for close monitoring, particularly in the setting of potentially dramatic fluid shifts. Blood loss may be high with transfusion required in most patients [14]. Special attention to thromboembolism prophylaxis, respiratory care, and nutrition is required. While no longer routine, some patients will require total parenteral nutrition due to prolonged inability to eat postoperatively, as ileus is relatively common [56]. A team-based approach, including case managers, dedicated nurses, and social workers, may help patients as they heal both mentally and physically postoperatively.

## 7. Quality of Life

As a portion of preoperative counseling and postoperative support, the changes in a woman's body image following PE must be reviewed. Some patients, particularly those undergoing this surgery for palliative management of pain or fistulae, do report improved quality of life following surgery, with decreased narcotic requirements and malodorous discharge [25]. Most women, however, note a decline in specific areas of quality of life. Most commonly sexual quality of life is diminished from preoperatively. Notably, body image, physical ability, and social function have all be reported decreased in questionnaires compared to patients' preoperative answers. These changes are more pronounced in younger patients and those who do not undergoing vaginal reconstruction. Interestingly, overall function and mental and emotional quality of life are comparable [57–59].

## 8. Conclusions

PE is a radical operation, involving en bloc resection of pelvic organs, including reproductive structures, bladder, and rectosigmoid. In gynecologic oncology, it is most commonly indicated for the treatment of advanced primary or locally recurrent cancer. Patients need to be carefully selected and counseled about risks and long-term issues related to the surgery. A comprehensive evaluation is required in order to exclude unresectable or metastatic disease. Total PE is associated with significant surgical morbidity, a fact that underscores the importance of careful patient selection and counseling. The emergence of minimally invasive surgery and application of this technology to radical pelvic surgery including PE may result in a reduction operative morbidity and mortality. Further studies are necessary prior to a widespread adoption of this technology to exenterative procedures.

## Figures and Tables

**Table 1:** Recent series of pelvic exenteration for gynecological malignancies.

Author	Year	*N*	Cervical	Uterine	Vulvar	Vaginal	Ovarian	Early complications	Late complications	Severe morbidity	Operative mortality	5-year survival
Benn et al. [60]	2011	54	40	9	5	0	0	50%	61%	44%		34%
Maggioni et al. [61]	2009	106	62	10	9	21	4	48%	49%		0%	
Marnitz et al. [12]	2006	55	55	0	0	0	0	11%	75%	38%	6%	37%
Goldberg et al. [11]	2006	103	95	2	1	0	0			25%	1%	47%
Sharma et al. [62]	2005	48	39	2	3	2	2	27%	75%	45%	4%	30%
Berek et al. [10]	2005	75	67*	8	0	*	0			23%	4%	54%

*Combined cervical and vaginal cancers.
